# Criterion Validity and Responsiveness of Estimated Cardiorespiratory Fitness Models in Patients with Inflammatory Joint Disease

**DOI:** 10.3390/jcm12216753

**Published:** 2023-10-25

**Authors:** Kristine Røren Nordén, Hanne Dagfinrud, Anne Grete Semb, Jonny Hisdal, George S. Metsios, Joseph Sexton, Camilla Fongen, Emilie Andrea Bakke, Anne Therese Tveter

**Affiliations:** 1 Center for Treatment of Rheumatic and Musculoskeletal Diseases (REMEDY), Norwegian National Advisory Unit on Rehabilitation in Rheumatology, Diakonhjemmet Hospital, 0319 Oslo, Norway; 2 Institute of Health and Society, Faculty of Medicine, University of Oslo, 0316 Oslo, Norway; 3 Norwegian National Unit for Rehabilitation for Rheumatic Patients with Special Needs, Diakonhjemmet Hospital, 0319 Oslo, Norway; 4 Preventive Cardio-Rheuma Clinic, Center for Treatment of Rheumatic and Musculoskeletal Diseases (REMEDY), Division of Rheumatology and Research, Diakonhjemmet Hospital, 0319 Oslo, Norway; 5 Institute of Clinical Medicine, Faculty of Medicine, University of Oslo, 0318 Oslo, Norway; jonny.hisdal@medisin.uio.no; 6 Department of Vascular Surgery, Oslo University Hospital—Aker, 0586 Oslo, Norway; 7 Department of Nutrition and Dietetics, University of Thessaly, 42132 Trikala, Greece; 8 Center for Treatment of Rheumatic and Musculoskeletal Diseases (REMEDY), Division of Rheumatology and Research, Diakonhjemmet Hospital, 0319 Oslo, Norway; 9 Faculty of Health Sciences, Institute of Rehabilitation Science and Health Technology, Oslo Metropolitan University, 0166 Oslo, Norway

**Keywords:** cardiorespiratory fitness, inflammatory joint disease, rheumatoid arthritis, spondyloarthritis, validity, responsiveness

## Abstract

Cardiorespiratory fitness (CRF) is an excellent marker of overall health. This study aimed to assess criterion validity and responsiveness of estimated CRF models (eCRF) in patients with inflammatory joint disease (IJD). CRF was measured directly as peak oxygen uptake (VO_2peak_) by a Cardiopulmonary Exercise Test (CPET), while one generic eCRF model (eCRF_GEN_) and two disease-specific eCRF models (eCRF_ALT_ and eCRF_PGA_) were used to estimate CRF at baseline and after 3 months in 55 Norwegian patients with IJD. Moderate correlations were observed between eCRF_GEN_, eCRF_ALT_, eCRF_PGA_, and VO_2peak_ at baseline (ICC 0.60, 0.64 and 0.62, respectively) and 3 months (ICC 0.62, 0.65 and 0.57, respectively). All eCRF models overestimated measured VO_2peak_, and there was large variability in agreement of individual measurements at baseline and at 3 months. Weak correlations were observed for responsiveness of eCRF_GEN_ (ICC 0.39), eCRF_ALT_ (ICC 0.40) and eCRF_PGA_ (ICC 0.39). Mean differences between change in eCRF models and change in VO_2peak_ were small, but the wide limits of agreement exceeded the pre-defined clinically acceptable margins. The eCRF models possessed adequate ability to detect ≥3.5 mL/kg/min improvement in VO_2peak_. eCRF may suffice for group-level assessment, but caution is advised when applying eCRF to individual patients with IJD.

## 1. Introduction

Cardiorespiratory fitness (CRF) reflects the functional capacity of the cardiovascular and respiratory systems to transport and deliver oxygen to working muscles during physical activity [1]. Comprehensive epidemiological data have shown consistent inverse associations between CRF and the risk of cardiovascular disease, cancer mortality, and all-cause mortality [2,3,4,5]. Furthermore, measures of CRF can offer valuable insights into the effectiveness of various interventions aimed at improving health outcomes. In light of this, CRF has gained recognition as an important indicator of overall health, and the American Heart Association advocates measures of CRF as a clinical vital sign [6].

Inflammatory joint diseases (IJD), including rheumatic diseases such as rheumatoid arthritis, spondyloarthritis, and psoriatic arthritis are characterized by joint inflammation, pain, fatigue, and varying levels of physical disability [7,8,9]. Additionally, IJD associates with an increased risk of CVD, influenced by systemic inflammation and a higher prevalence of classic CVD risk factors [10,11,12]. There are multiple observations of inferior levels of CRF in patients with rheumatoid arthritis [13,14], spondyloarthritis [15,16], and combined diagnoses [17], and low levels of CRF may contribute to the elevated CVD and mortality risk [18,19]. Accurate assessment of CRF is therefore of clinical value in IJD care, as it can shed light on CVD risk and measure the effects of interventions designed to enhance CRF.

The criterion method to assess CRF is the Cardiopulmonary Exercise Test (CPET), which measures peak oxygen uptake (VO_2peak_) by indirect calorimetry during a progressive exercise test to maximal exertion [1,6]. However, CPET is time-consuming and requires specialized equipment and trained personnel, thereby restricting its applicability to large-scale studies and primary care settings [6]. To overcome these limitations and facilitate broader implementation of CRF assessment, researchers have explored the potential of non-exercise estimated CRF (eCRF) algorithms. These models often apply age, gender, and a measure of body composition, combined with self-reported habitual exercise and/or physical activity, to estimate an individual’s CRF without the need for an exercise test [6]. Among the available eCRF algorithms, a generic model developed in a Norwegian population [20,21] has been recommended for use [22]. In an attempt to address characteristics of patients with rheumatoid arthritis that may associate with CRF, the original eCRF model [20] has been adapted in two rheumatoid arthritis-specific models [23].

A trend to overestimate CRF in individuals at the lower end of the fitness spectrum is reported across various eCRF models [22]. As the accuracy of eCRF may vary depending on the characteristics of the population studied, it is essential to verify the validity of eCRF models in target populations with underlying health conditions. Before incorporating eCRF in IJD settings, it is equally important to assess the ability of eCRF models to detect positive changes in CRF resulting from a more active lifestyle, as well as the ability to capture declining levels of CRF that may call for additional assessments and referral to exercise interventions. Despite the simplicity of eCRF, the responsiveness, i.e., longitudinal validity [24], of these models remains largely unknown [21], and requires further investigation. Accordingly, the purpose of the current study was to evaluate the criterion validity and responsiveness of eCRF compared to the gold standard VO_2peak_ among patients with IJD. We hypothesized that, at the group level, eCRF models would demonstrate a tendency to overestimate measured VO_2peak_ and change in VO_2peak_.

## 2. Materials and Methods

### 2.1. Patients and Study Setting

Data from 55 Norwegian patients who attended baseline and 3-month study visits in the ExeHeart randomized controlled trial (RCT) (ClinicalTrials.gov NCT04922840) were included in the present analyses. Approval of the ExeHeart trial, including the current study, was granted by the Regional Committee for Medical and Health Research Ethics (201227) and the Data Protection Officer at Diakonhjemmet Hospital (reg.no. 00397). Study procedures adhered to the principles of the Helsinki Declaration and all patients provided informed written consent.

Details regarding the study sample, patient enrollment and outcome assessment are fully described in the ExeHeart study protocol [25]. In short, the aim of the ExeHeart trial was to assess the effect of 12 weeks of high-intensity interval training on CRF, classic cardiovascular disease risk factors and disease activity in patients with IJD. Trial results are being published elsewhere. The study enrolled individuals that met the following criteria: aged 18–70 years, with a body mass index (BMI) ranging from 18.5 to 40, IJD diagnosis (rheumatoid arthritis, spondyloarthritis or psoriatic arthritis), capable of walking unaided for ≥15 min, and proficient in Norwegian and/or English. Patients with lower-extremity injury or surgery within the last 12 months, primary neurological disease, cognitive impairment, engagement in high intensity exercise in the three months prior to study inclusion and/or contraindications to maximal exercise testing [1] were excluded from study participation. Although no formal power calculation was conducted specifically for the current study, a sample size of 55 patients aligns with the recommended number for criterion validity approach in COnsensus-based Standards for the selection of health Measurement INstruments (COSMIN) guidelines [26]. The study was reported according to Guidelines for Reporting Reliability and Agreement Studies [27] and the COSMIN Reporting guideline for studies on measurement properties of patient reported outcome measures [28].

### 2.2. Outcome Assessment and Interim Period

Study visits took place at Diakonhjemmet Hospital, Oslo, Norway, from August 2021 to November 2022. During the interim period from baseline assessment to the 3-month session, 27 out of the 55 patients participated in a 12-week high-intensity interval training program (exercise group), while the remaining 28 patients formed the control group and did not receive any targeted exercise intervention. The 3-month sessions were scheduled at the same time of day as the corresponding baseline sessions. Outcome assessors (KRN, CF, and EAB) were physiotherapists with extensive experience in rheumatology and exercise testing, and were blinded to group allocation.

### 2.3. Demographic Variables

In the week leading up to study visits, patients answered a digital questionnaire [25], and items regarding education level, medication usage, and pain and fatigue over the past week (numerical rating scale 0–10, 0 = best) were included to describe the study sample, along with the following: IJD diagnosis and comorbidities were extracted from the patient’s medical record. IJD-specific composite measures were applied to measure clinical disease activity, and disease activity was further categorized as remission, low, moderate or high using instrument-specific cut-off values. Further examinations included blood chemistry (lipid profile, C-reactive protein and erythrocyte sedimentation rate) and blood pressure measurements [25].

### 2.4. Criterion Measurement of CRF

CRF was measured directly as VO_2peak_ in mL/kg/min by the criterion method CPET [6,29]. Test equipment was calibrated according to the manufacturer’s specifications with gas calibration every third hour, and automatic calibration of volume sensors between each test. Accuracy of the volume sensors was confirmed weekly through manual calibration with a 3 L syringe (Hans Rudolph Inc., Shawnee, KS, USA).

Patients were instructed to have a two-hour fast since their last meal, avoid the use of nicotine and caffeine for at least four hours prior to the test, and refrain from participating in vigorous exercise within the 24 h preceding the CPET. Pretest spirometry and maximal voluntary ventilation were conducted in accordance with recommendations [29,30]. The CPET was performed on a treadmill (PPS 55 Woodway, Würzburg, Germany), with 12-lead electrocardiogram (Customed cardio 300 BT_A, CareFusion, Ottobrunn, Germany), blood pressure monitor (Suntech Tango M2, SunTech Medical, Morrisville, NC, USA), and earlobe pulse oximetry. Ventilatory parameters and gas exchange data were collected breath-by-breath using a Hans Rudolph two-way mask (7450 series, Hans Rudolph Inc., Shawnee, KS, USA), analyzed by a metabolic cart (Vyntus CPX, Vyaire Medial, Hoechenberg, Germany), and balanced over 30 s intervals. A modified Balke ramp protocol [31] was applied, starting with a short warm-up period to familiarize the patient with the treadmill and determine an appropriate initial walking speed. Following warm-up, inclination was increased by 2% every minute up to 15 or 20%. If the patient was able to continue, speed was increased by 0.5 km/h per minute until volitional exhaustion. Ventilatory reserve was calculated as (maximal minute ventilation-peak minute ventilation/maximal minute ventilation) × 100 [29]. The criteria for VO_2peak_ was a visible plateau in oxygen uptake despite an increase in work load. Notably, such plateaus are not always observed in patient populations [32], and in the absence of a VO_2_ plateau, acceptable criteria for VO_2peak_ by CPET were defined as meeting ≥2 of the following criteria [30]: (a) peak heart rate within 90% of predicted (220—age), (b) Borg rating of perceived exertion ≥9 (0–10, 10 = maximal exertion), (c) ventilatory reserve less than 15% of maximal minute ventilation, and/or (d) adherence to age- and gender-specific cut-off values for respiratory exchange ratio and post-exercise blood lactate [32]. 

### 2.5. Estimated CRF

The generic eCRF (eCRF_GEN_) model was used in all patients and includes the variables age, BMI, resting heart rate, and physical activity index [20,21].

Body mass to the nearest 0.1 kg (Tanita MC-780MA Tanita Corporation, Tokyo, Japan) and height to the nearest cm (KaWe Person Check, Kirchner & Wilhelm GmbH + Co. KG, Asperg, Germany) were used to calculate BMI as kg/m^2^. 

Resting heart rate (beats/min) was measured after 10 min of rest in a supine position using a mobile blood pressure monitor (Mobil-o-graph PWA, I.E.M. GmbH, Stolberg, Germany) and recorded as the mean of two measurements. 

From the digital questionnaire, three items regarding exercise frequency, duration and intensity were used to calculate a physical activity index ranging from 0 to 45, with higher scores indicating better physical activity levels [20,33]. 

In addition to the variables included in eCRF_GEN_, there are two rheumatoid arthritis-specific models [23]. The first model, eCRF_ALT_, includes self-reported smoking status and was explored in all patients regardless of IJD entity. The second model, eCRF_PGA_, further incorporates Patient Global Assessment of disease activity (PGA), and was answered by patients with rheumatoid arthritis in our study sample. For the purpose of scrutinizing these models, smoking status was coded as 1 for “ever smoked” or “current smoker” and 0 for “never smoked” [23]. PGA was phrased as “Considering all the symptoms from your rheumatic disease during the last week, how do you think your state is?” and answered on a 0–100 mm visual analog scale with 0 anchored as “good, no symptoms” and 100 anchored as “very severe” [23]. Equations for eCRF_GEN_, eCRF_ALT_ and eCRF_PGA_ are provided in Table 1. 

### 2.6. Statistical Analysis

Continuous data are presented as mean with standard deviation (SD) or median with interquartile range (IQR) for skewed data, whereas counts with percentages are provided for categorical data. Paired sample *t*-tests were used to calculate differences between corresponding measures of CRF at baseline and at 3 months, as well as between the individual eCRF models and VO_2peak_. Intraclass correlation coefficient (ICC) two-way mixed-effects models with 95% confidence interval (CI) were used to assess absolute agreement between eCRF models and VO_2peak_ at baseline and 3 months, and change from baseline to 3 months. An ICC value <0.5 indicates poor agreement, 0.5–0.75 moderate agreement, 0.75–0.9 good agreement and values >0.9 excellent agreement [34]. A limitation of using correlation to assess responsiveness involves narrowing of the variable range, as this will often lead to lower correlation coefficients [35,36]. Therefore, we conducted supplementary univariate linear regression analyses with change in VO_2peak_ as the dependent variable and change in the respective eCRF model as the independent variable. Resulting regression coefficients offer insight into predicting change in VO_2peak_ following a one-unit increase in eCRF, while the R^2^ statistic gives the fraction of change in VO_2peak_ explained by a unit change in eCRF. Model assumptions were assessed graphically. 

Bland–Altman plots were generated to visualize the relationship between eCRF models and VO_2peak_ at baseline and 3 months, as well as the change in eCRF models and VO_2peak_ from baseline to 3 months. Plots include the average of eCRF models and VO_2peak_ on the *x*-axis, difference between the two methods on the *y*-axis, mean bias and 95% limits of agreement [37]. Assumptions of normal distribution of differences were checked by histograms and Shapiro–Wilk tests [38]. A 3.5 mL/kg/min increase in CRF associates with a significant reduction in cardiovascular disease and mortality risks [39,40], and a range of ±3.5 mL/kg/min was defined as the clinically acceptable difference between measurement methods. 

We further investigated the capability of eCRF models to detect improvement in CRF using area under the curve (AUC) obtained from empirical receiver operating curve (ROC) analysis. A threshold of ≥3.5 mL/kg/min was used to dichotomize a substantial improvement in VO_2peak_ from baseline to 3 months, and AUC ≥ 0.80 was deemed acceptable [35,41]. As there were only two observations of a CRF decline ≥ 3.5 mL/kg/min within the patient cohort, the available dataset did not facilitate ROC analysis of the ability of eCRF models to accurately identify substantial deteriorations in VO_2peak_. 

A significance threshold of *p* < 0.05 was applied for all analyses, and STATA v. 17 was used for all statistical computations.

## 3. Results

### 3.1. Demographic and Clinical Characteristics

A total of 55 patients (31 women and 24 men) with IJD, with a median age of 59 years (IQR 51–63), and a median BMI of 25 (IQR 22–31) were included. Clinical characteristics from baseline sessions are provided in Table 2 and Supplementary Files, Table S1. Mean (range) VO_2peak_ was 30.3 (17.3, 47.9) mL/kg/min at baseline and 31.6 (18.9, 49.3) mL/kg/min at 3 months. Mean (range) change in VO_2peak_ from baseline to 3 months was 1.3 (−5.3, 7.8) mL/kg/min, and 13 (24%) patients exhibited a ≥3.5 mL/kg/min improvement in VO_2peak_ (Table 3 and Supplementary Files, Table S2). 

### 3.2. ICC Analysis

As detailed in Table 3, measured VO_2peak_ and eCRF models were moderately correlated, with ICC values ranging from 0.60 to 0.64 at baseline and 0.57 to 0.65 at 3 months. Regarding change from baseline to 3 months, correlation coefficients were lower, ranging from 0.39 to 0.40 between changes in eCRF models and changes in VO_2peak_. In the complemental regression analysis, individual regression coefficients were statistically significant. An increment of one unit in eCRF_GEN_ associated with a change of 0.56 mL/kg/min in VO_2peak_. Corresponding coefficients for change in eCRF_ALT_ and eCRF_PGA_ indicated an associated change of 0.58 and 0.68 mL/kg/min, respectively, in VO_2peak_. The proportion of variation in VO_2peak_ change explained by change in eCRF (R^2^) varied from 17 to 24%.

### 3.3. Bland–Altman Analysis

**Figure 1:**
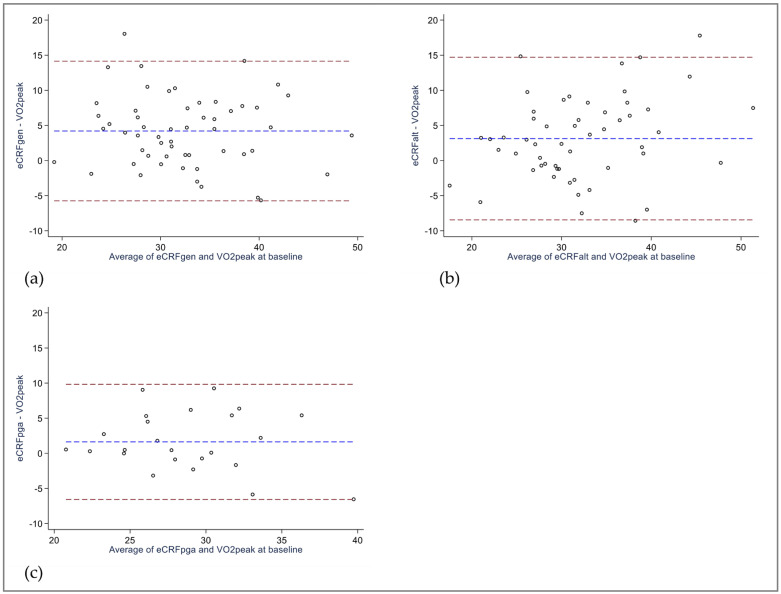
BlandAltman plots of eCRF versus VO_2peak_ at **baseline** (**a**) eCRF_GEN_ vs. VO_2peak_, *n* = 55 (**b**) eCRF_ALT_ vs. VO_2peak_, *n* = 55 (**c**) eCRF_PGA_ vs. VO_2peak_, *n* = 24. Blue dotted line illustrates mean bias, red dotted lines illustrate 95% limits of agreement. eCRF_ALT:_ Alternative rheumatoid arthritis-specific eCRF model. eCRF_GEN_: Estimated cardiorespiratory fitness, generic model. eCRF_PGA_: Rheumatoid arthritis-specific eCRF model, assessed in patients with rheumatoid arthritis. VO_2peak_: Peak oxygen uptake.

At baseline, eCRF overestimated VO_2peak_ with a significant mean bias of 4.2 mL/kg/min for eCRF_GEN_ and 3.1 mL/kg/min for eCRF_ALT_. Conversely, the 1.6 mL/kg/min difference observed in the subsample analyzed using eCRF_PGA_ was not statistically significant (Table 3). The 95% limits of agreement showed large variability of agreement between eCRF models and VO_2peak_, and exceeded the clinical threshold of ±3.5 mL/kg/min (Figure 1a–c). 

**Figure 2:**
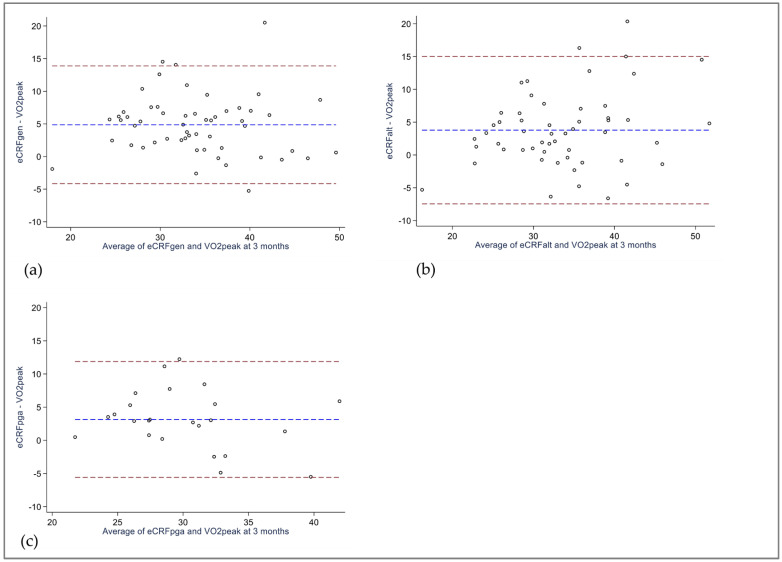
BlandAltman plots of eCRF versus VO_2peak_ at 3 months (**a**) eCRF_GEN_ vs. VO_2peak_, *n* = 55 (**b**) eCRF_ALT_ vs. VO_2peak_, *n* = 54 (**c**) eCRF_PGA_ vs. VO_2peak,_ *n* = 24. Blue dotted line illustrates mean bias, red dotted lines illustrate 95% limits of agreement. eCRF_ALT:_ Alternative rheumatoid arthritis-specific eCRF model. eCRF_GEN_: Estimated cardiorespiratory fitness, generic model. eCRF_PGA_: Rheumatoid arthritis-specific eCRF model, assessed in patients with rheumatoid arthritis. VO_2peak_: Peak oxygen uptake.

For the 3-month measurements, all eCRF models overestimated VO_2peak_, with a significant mean bias of 4.9 mL/kg/min for eCRF_GEN_, 3.8 mL/kg/min for eCRF_ALT_, and 3.1 mL/kg/min for the subsample scrutinized using eCRF_PGA_ (Table 3). As reflected in Figure 2a–c, the 95% limits of agreement surpassed the clinical threshold. 

**Figure 3:**
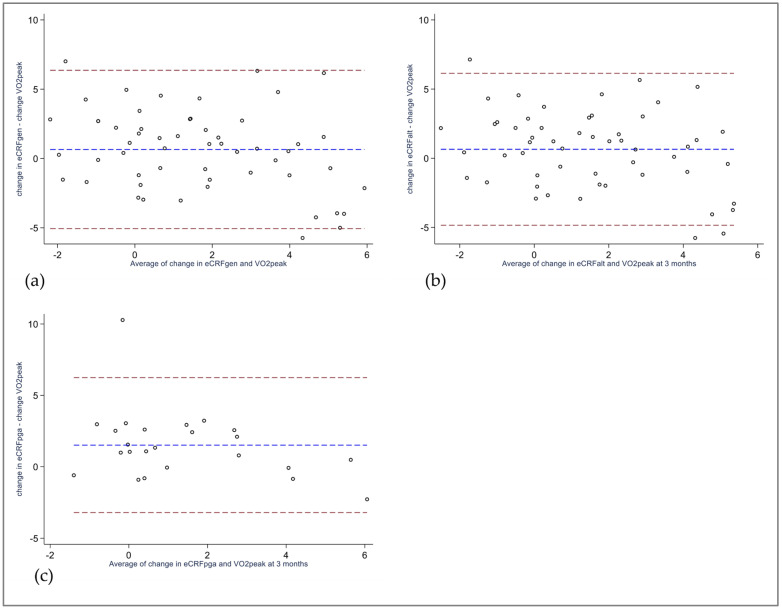
Bland–Altman plots of change in eCRF versus change in VO_2peak_ from baseline to 3 months (**a**) eCRF_GEN_ vs. VO_2peak,_ *n* = 55 (**b**) eCRF_ALT_ vs. VO_2peak_, *n* = 54 (**c**) eCRF_ALT_ vs. VO_2peak_, *n =* 24. Blue dotted line illustrates mean bias, red dotted lines illustrate 95% limits of agreement. eCRF_ALT:_ Alternative Rheumatoid Arthritis-specific eCRF model. eCRF_GEN_: Estimated cardiorespiratory fitness, generic model. eCRF_PGA_: Rheumatoid arthritis-specific eCRF model, assessed in patients with rheumatoid arthritis. VO_2peak_: Peak oxygen uptake.

Regarding change scores from baseline to 3 months, both eCRF_GEN_ and eCRF_ALT_ revealed non-significant mean biases of 0.7 mL/kg/min, while the subsample analyzed using eCRF_PGA_ demonstrated a significant mean bias of 1.5 mL/kg/min (Table 3). The 95% limits of agreement exceeded the clinically acceptable difference (Figure 3a–c). 

### 3.4. Area-under-the-Curve Analysis

In order to assess the performance of eCRF models in predicting a threshold improvement in VO_2peak_, patients with a decline in VO_2peak_ of ≥3.5 mL/kg/min from baseline to 3 months were excluded. ROC curves are presented in Supplementary Files, Figure S1. Among the 53 patients analyzed using eCRF_GEN_, 13 patients had an improvement in VO_2peak_ ≥ 3.5 mL/kg/min, and AUC for eCRF_GEN_ was 0.82 (95% CI 0.71 to 0.93). In the 52 patients assessed using eCRF_ALT_ (one excluded due to incomplete eCRF data at 3 months), 12 patients demonstrated an improvement ≥3.5 mL/kg/min in VO_2peak_, and AUC for eCRF_ALT_ was 0.83 (95% CI 0.72 to 0.94). Within the subsample of 23 patients evaluated using eCRF_PGA_, 4 patients had a ≥3.5 mL/kg/min improvement in VO_2peak_, and eCRF_PGA_ yielded an AUC of 0.97 (95% CI 0.91 to 1.00). 

## 4. Discussion

Growing recognition of the value of CRF as a robust indicator of overall health has emphasized the need for valid and practical measures of CRF. The present study evaluated the validity and responsiveness of eCRF models in comparison to the criterion measure VO_2peak_ among patients with IJD. Our key findings revealed moderate correlations between eCRF models and VO_2peak_, weak correlations between change scores, and large variability in agreement of individual measurements. Notably, all eCRF models demonstrated adequate performance in identifying larger improvements in VO_2peak_.

In our data, we observed moderate agreement between eCRF and VO_2peak_, indicating that while eCRF models may not precisely mirror VO_2peak_, they can still capture a considerable correlation between these two measures of CRF among patients with IJD. These findings align with three other studies that have investigated the accuracy of several eCRF models in healthy adults and older individuals [5,22,42]. Collectively, this highlights a potential use for eCRF models to estimate VO_2peak_ at a group level. However, our Bland–Altman plots from baseline and 3 months into the study revealed wide limits of agreement that exceeded our pre-defined clinically acceptable difference. This observation parallels reports of an extensive range in the difference between eCRF and VO_2peak_ in other study samples [5,22,43]. Articles detailing the development of the eCRF models currently under investigation report a tendency to overestimate CRF, especially in individuals with VO_2peak_ below 30 mL/kg/min [21,23]. In line with this, our own study data with mean VO_2peak_ values of around 30–31 mL/kg/min demonstrated a consistent trend, where both eCRF_GEN_ and eCRF_ALT_ overestimated VO_2peak_ at baseline and all eCRF models overestimated VO_2peak_ at 3 months. Collectively, this illustrates that eCRF models may not perform well in individual patients, and underlines the need for caution in applying eCRF to patients with presumably low fitness levels. 

Assessing responsiveness lends insight into a measurement tool’s capability to detect longitudinal change, and we believe the research design used herein allows us to draw inferences about the eCRF models’ ability to capture change in VO_2peak_. Our data demonstrated poor correlation between changes observed in the eCRF models and changes in VO_2peak._ However, due to inherent restraints when examining change scores, lower correlation coefficients were anticipated. Beta coefficients derived from the regression analyses suggest that, depending on the specific eCRF model applied, a one-unit increase in eCRF corresponded to a change in VO_2peak_ ranging from 0.56 to 0.68 mL/kg/min. Along with the modest proportions of change in VO_2peak_ predicted by change in eCRF (R^2^), this implies that eCRF models have weak ability to predict a true change in CRF. Although the mean differences between change captured by eCRF models and change in VO_2peak_ were, for the most part, small and statistically non-significant, the Bland–Altman limits of agreement extended beyond the range considered clinically acceptable. Few other studies have explored the longitudinal validity of eCRF models. Lannoy and Ross [44] examined the same eCRF_GEN_ model used in our study within an RCT where participants were randomized to exercise at various intensity levels. Apart from participants randomized to high intensity exercise, no significant group differences were observed between changes in VO_2peak_ and eCRF at any timepoint. However, the authors of said study emphasized the presence of wide limits of agreement. Coupled with our results from the present study, this underscores that measures of change in eCRF and change in VO_2peak_ are not interchangeable at an individual level. 

Epidemiological studies that examine a risk threshold of 3.5 mL/kg/min often rely on indirect measurement techniques such as eCRF and self-reported exercise. As discussed by Lannoy and Ross [44], within eCRF models, elements such as age and gender typically remain constant, while factors like BMI and resting heart rate might show small short-term variations. Accordingly, short-term changes in eCRF are largely reliant on shifts in self-reported physical activity behavior, suggesting that well-designed exercise interventions that improve CRF should be detectable using eCRF. However, several studies have demonstrated substantial variations in individual change in VO_2peak_ in response to a standardized exercise program, often attributed to differences in training dose, adherence to exercise, and genetic differences in exercise response [45]. eCRF models may struggle to capture individual response to exercise and will apply uniform change in CRF in response to change in the variables included in the model. In turn, physical activity may be prone to misclassifications, as the physical activity index in the current eCRF analyses relies heavily on self-reported intensity of exercise, and self-reported physical activity may be influenced by desirability bias [46,47]. Moreover, in the eCRF_PGA_ model, disease activity (PGA) may correlate with changes in physical activity, considering that elevated disease activity associates with lower levels of physical activity [48]. In contrast to eCRF, CPET provides a precise measure of VO_2peak_, with even a 1 mL/kg/min improvement linked to reduced risk of cardiovascular disease and mortality [3]. Although achieving the same level of accuracy from eCRF may be unrealistic, these models should be able to uncover larger changes in CRF. Our AUC estimates suggest that eCRF_GEN_ and eCRF_ALT_ models can correctly identify >80% of the patients with a ≥3.5 mL/kg/min improvement in VO_2peak_. The eCRF_PGA_ model performed even better, identifying 97% of the patients. Notably, the performance of the eCRF_PGA_ model was tested in a small sample of patients, and caution is advised when interpreting these results. Furthermore, as only two patients in our cohort demonstrated ≥3.5 mL/kg/min deterioration in VO_2peak_, the performance of eCRF models in capturing larger deteriorations among IJD patients remains unknown, and requires further investigation. 

### 4.1. Clinical Implications and Future Research Avenues

Although eCRF has limitations in accuracy, the low cost and user-friendliness make it an appealing option. For instance, eCRF sidesteps the need for a maximal exercise test by using self-reported, easily accessible data, whereas VO_2peak_ assessed using CPET may exclude individuals with contraindications to maximal exercise tests or physical limitations [1], thereby resulting in the absence of CRF measures for these patients. Moreover, CRF obtained using eCRF is inversely related to cardiovascular disease and all-cause mortality [5,21,49], which adds valuable prognostic information. It may be important to distinguish between using eCRF in research and clinical contexts. For research that focuses on group trends, eCRF may be sufficient. In clinical care, eCRF may hold value as an initial screening tool to identify individual patients with low CRF that warrant more comprehensive tests such as CPET. However, clinicians need to be aware of the tendency for eCRF to overestimate CRF, especially in individuals with poor fitness levels [6,22,42], as this can have implications for risk interpretation and clinical assessment. Using eCRF in clinical practice as a surrogate for VO_2peak_ may therefore require a careful weighing of convenience versus limitations in accuracy. Given that CRF serves as a valuable measure of overall health, the potential benefits of eCRF may outweigh the inaccuracies, as having a rough CRF measure is better than none at all. 

A path for future research could be to investigate whether adding eCRF models to cardiovascular disease risk assessment can improve the ability to predict cardiovascular disease and mortality in patients with IJD. Additionally, indirect tests that use variables such as workload and heart rate from maximal or submaximal exercise tests to estimate VO_2peak_ are considered second-best to CPET [1]. Assessing the validity of indirect exercise tests in modern-day IJD populations can offer insights into their potential as clinically feasible estimates of VO_2peak_. 

### 4.2. Strengths and Limitations

The primary strengths of the present study lie in our application of COSMIN recommendations to assess criterion validity and responsiveness, and the use of CPET to measure CRF. However, several limitations need to be addressed. Subtle biological variations and lack of consensus regarding the use of end criteria [29,30] illustrate that while CPET is recognized as the gold standard to measure CRF, the interpretation of test results is not an exact science. A treadmill was used in all CPETs in our sample, and our results may not extend to comparing eCRF models with VO_2peak_ from CPETs with a cycle ergometer. Our study cohort included participants from an RCT, and potential selection bias may limit the applicability of study results to the general IJD population. Caution is also advised in extrapolating results from the present study to different demographic groups and other eCRF models, as our cohort included individuals from Norway and the eCRF models under scrutiny were derived from Norwegian study samples. Lastly, the eCRF_PGA_ model was evaluated in a small subset of patients, and results should be viewed as exploratory. 

## 5. Conclusions

In summary, eCRF models demonstrated moderate criterion validity, suggesting a potential group-level applicability in patients with IJD. However, caution is advised in adopting eCRF for individual patients, due to the wide limits of agreement and a tendency to overestimate true CRF. eCRF models are less suited to measure change at the individual level, although these models have adequate ability to detect larger improvements in VO_2peak_ among patients with IJD. Balancing convenience with limitations in accuracy of individual measurements is crucial before applying eCRF models in research and clinical settings.

## Figures and Tables

**Table 1:** Equations for the generic and RA-specific eCRF models.

Model	eCRF Equation
eCRF_GEN_ [21] *Female* *Male*	70.77 − (0.244 × age) − (0.749 × BMI) − (0.107 × resting heart rate) + (0.213 × physical activity index) 92.05 − (0.327 × age) − (0.933 × BMI) − (0.167 × resting heart rate) + (0.257 × physical activity index)
eCRF_ALT_ [23] eCRF_PGA_ [23]	(female = 0, male = 1; never smoked = 0, ever smoked = 1) 77.851 + (gender × 25.460) − (age × 0.381) − (age–gender interaction × 0.254) − (BMI × 0.743) − (resting heart rate × 0.115) − (smoking × 2.154) + (physical activity index × 0.209) 77.961 + (gender × 28.791) − (age × 0.358) − (age–gender interaction × 0.326) − (BMI × 0.700) − (resting heart rate × 0.125) − (smoking × 1.854) + (physical activity index × 0.211) − (PGA × 0.071)

BMI: Body mass index, eCRF_ALT:_ Alternative rheumatoid arthritis-specific eCRF model, eCRF_GEN_: Estimated cardiorespiratory fitness, generic model, eCRF_PGA_: Rheumatoid arthritis-specific eCRF model, PGA: Patient global assessment of disease activity.

**Table 2:** Clinical characteristics from baseline sessions (*n* = 55).

Variable	
Age, years, median (IQR)	59 (51–63)
Gender, female, *n* (%)	31 (56)
Education > 12 years, *n* (%)	42 (76)
Anthropometrics Height, cm, mean (SD) Weight, kg, median (IQR) BMI, median (IQR)	173 (9) 74 (66–91) 25 (22–31)
Diagnosis Rheumatoid arthritis, *n* (%) Spondyloarthritis, *n* (%) Psoriatic arthritis, *n* (%)	26 (47) 16 (29) 13 (24)
IJD disease duration, years, median (IQR)	15 (7–30)
Disease activity categorized Remission, *n* (%) Low, *n* (%) Moderate, *n* (%) High, *n* (%)	21 (38) 15 (27) 12 (22) 7 (13)
Comorbidity Diabetes, *n* (%) Chronic obstructive pulmonary disease, *n* (%) Inflammatory bowel disease, *n* (%)	3 (5) 4 (7) 4 (7)
Ever smoker, yes, *n* (%)	34 (62)
Resting heart rate, beats/min, mean (SD)	68 (11)
Cardiopulmonary Exercise Test VO_2peak_, mL/kg/min, mean (SD) VO_2peak_, L/min, mean (SD) VO_2_ plateau at peak exercise, yes, *n* (%) Respiratory exchange ratio, VCO_2_/VO_2_, mean (SD) Borg RPE 0–10 (10 = maximal), median (IQR) Peak heart rate, beats/min, mean (SD) Percent of predicted peak heart rate (220-age), mean (SD) Post-exercise blood lactate, mmol/L, median (IQR) Ventilatory reserve, %, mean (SD)	30.3 (6.9) 2.4 (0.7) 29 (53) 1.16 (0.1) 10 (9–10) 165 (14) 101 (7) 9.2 (7.3–11.7) § 25 (13) ^
Physical activity index (0–45, 45 = best), median (IQR)	0 (0–15)
Numerical Rating Scale (0–10), 0 = best Pain, median (IQR) Fatigue, median (IQR)	2 (1–4) 3 (1–5)
Patient Global Assessment, 0–100 mm, 0 = best, mean (SD)	29 (22) $

§ *n* = 52; values ≥20 mmol/L omitted. ^ *n* = 54 patients. $ *n* = 24 patients with rheumatoid arthritis. BMI: Body Mass Index, IJD: Inflammatory Joint Disease, RPE: Rating of Perceived Exertion. VCO_2_: Volume of carbon dioxide production. VO_2_: Volume of oxygen uptake. VO_2peak_: Peak oxygen uptake.

**Table 3:** Agreement between VO_2peak_ and eCRF models at baseline, 3 months, and change from baseline to 3 months. Values are presented as mean (SD) unless otherwise indicated.

Variable	Baseline (*n =* 55)	3 Months (*n =* 55)	∆Baseline to 3 Months (*n =* 55)
VO_2peak_, mL/kg/min	30.3 (6.9)	31.6 (7.0)	1.3 (0.5 to 2.1) ^a^
eCRF_GEN_, mL/kg/min ICC (95% CI) Linear regression, regression coefficient, (95% CI) R2 Difference eCRF_GEN_—VO_2peak_, mL/kg/min. (95% CI) 95% Limits of agreement	34.5 (6.5) 0.60 (0.15 to 0.80) 4.2 (2.8 to 5.6) ^a^ −5.7 to 14.1	36.4 (6.7) 0.62 (0.03 to 0.84) 4.9 (3.6 to 6.1) ^a^ −4.2 to 13.9	2.0 (1.4 to 2.6) ^a^ 0.39 (0.15 to 0.59) 0.56 (0.22 to 0.90) 0.17 0.7 (−0.1 to 1.4) ^a^ −5.1 to 6.4
eCRF_ALT_, mL/kg/min ICC (95% CI) Linear regression, regression coefficient, (95% CI) R2 Difference eCRFALT—VO_2peak_, mL/kg/min, (95% CI) 95% Limits of agreement	33.6 (8.3) 0.64 (0.38 to 0.79) 3.1 (1.5 to 4.7) ^a^ −8.4 to 14.7	35.5 (8.4) § 0.65 (0.31 to 0.82) § 3.8 (2.2 to 5.3) §^a^ −7.5 to 15.0 §	1.8 (1.2 to 2.4) §^a^ 0.40 (0.16 to 0.60) § 0.58 (0.24 to 0.91) § 0.19 § 0.7 (−0.1 to 1.4) §^a^ −4.8 to 6.1 §
**Variable**	**Baseline (*n* = 24)**	**3 months (*n* = 24)**	**∆Baseline to 3 months (*n* = 24)**
VO_2peak,_ mL/kg/min	27.9 (5.2)	28.6 (5.8)	0.6 (−0.5 to 1.8) ^a^
eCRF_PGA_, mL/kg/min ICC (95% CI) Linear regression, regression coefficient, (95% CI) R2 Difference eCRFPGA—VO_2peak_, mL/kg/min, (95% CI) 95% Limits of agreement	29.6 (4.6) 0.62 (0.30 to 0.81) 1.6 (−0.1 to 3.4) ^a^ −6.6 to 9.8	31.7 (4.8) 0.57 (0.13 to 0.81) 3.1 (1.3 to 5.0) ^a^ −5.6 to 11.9	2.1 (1.3 to 3.0) ^a^ 0.39 (0.01 to 0.68) 0.68 (0.06 to 1.29) ^b^ 0.24 1.5 (0.5 to 2.5) ^a^ −3.2 to 6.2

^a^ Analyzed by paired samples *t*-test. ^b^ Bootstrap confidence intervals, 50 replications. ∆ = Change. § *n* = 54 patients (smoking status missing at 3 months; *n* = 1). eCRF_ALT:_ Alternative rheumatoid arthritis-specific eCRF model. eCRF_GEN_: Estimated cardiorespiratory fitness, generic model. eCRF_PGA_: Rheumatoid arthritis-specific eCRF model, assessed in patients with rheumatoid arthritis. ICC: Intraclass Correlation Coefficient. VO_2peak_: Peak oxygen uptake.

## Data Availability

Data can be shared upon reasonable request to the corresponding author.

## Supplementary Materials

The following supporting information can be downloaded at: https://www.mdpi.com/article/10.3390/jcm12216753/s1, Figure S1: ROC curves evaluating the ability of eCRF models to predict ≥3.5 mL/kg/min improvement in VO_2peak. from_ baseline to 3 months; Table S1: Additional clinical characteristics from baseline sessions; Table S2: Cardiopulmonary Exercise Test characteristics from 3-month study visits.
