# Supplementary material for: Criterion Validity and Responsiveness of Estimated Cardiorespiratory Fitness Models in Patients with Inflammatory Joint Disease
Source: J Clin Med. 2023 Oct 25;12(21):6753. doi: 10.3390/jcm12216753 (PMC10650593; doi:10.3390/jcm12216753)
Supplement: Supplementary file 1 [file jcm-12-06753-s001.zip › jcm-2652547-supplementary.pdf]

Supplementary Materials

Criterion Validity and Responsiveness of Estimated Cardiorespiratory Fitness Models in Patients with Inflammatory Joint Disease

Table S1. Additional clinical characteristics from baseline sessions.

**Variable (*n*=55)**	
Anthropometric measures	
Waist circumference, cm	93 (13)
Fat mass, kg, median (IQR)§	19 (14-27)
Fat-free mass, kg §, median (IQR)	52 (45-64)
Visceral fat indicator, median (IQR)§	8 (5-11)
Inflammatory markers	
CRP, mg/L, median (IQR)	1 (1-2)
ESR, mm, median (IQR)§	9 (5-15)
IJD medication	
Conventional DMARDs, n (%)	24 (44)
Biologics and/or JAK inhibitors, n (%)	38 (69)
Cortisone, n (%)	11 (20)
NSAIDs, n (%)	32 (58)
Analgesics	
Non-opioids, n (%)	38 (69)
Weak opioids, n (%)	5 (9)
Strong opioids, n (%)	0 (0)
Blood pressure	
Systolic, mmHg	127 (13)
Diastolic, mmHg	83 (9.7)
Mean arterial pressure, mmHg	103 (10)
Lipids	
Total cholesterol, mmol/L, mean (SD)	4.8 (1.3)
HDL-c, mmol/L, median (IQR)	1.5 (1.2-2.0)
LDL-c, mmol/L, median (IQR)	2.4 (1.6-3.4)
Triglycerides, mmol/L, median (IQR)	1.3 (0.8-1.7)
CVD medication	
Statins, n (%)	32 (58)
Antihypertensives, n (%)	10 (18)
Betablockers, n (%)	1 (2)

§ *n*=54. CRP: C-reactive protein, CVD: cardiovascular disease, DMARDs: disease-modifying anti-rheumatic drugs, ESR: erythrocyte sedimentation rate, HDL-c: high-density lipoprotein cholesterol, IJD: Inflammatory Joint Disease, JAK: Janus Kinase inhibitors, LDL-c: low-density lipoprotein cholesterol, NSAIDs: non-steroidal anti-inflammatory drugs

Table S2. CardioPulmonary Exercise Test Characteristics from 3-month study visits. Values are presented as mean (SD) unless otherwise indicated.

**Variable**	**3 months (*n*=55)**
VO_{2peak}, mL/kg/min	31.6 (70)
VO_{2peak}, L/min	2.5 (0.7)
VO_2 plateau at peak exercise, yes, n (%)	29 (53)
Respiratory exchange ratio, VCO_2/VO_2	1.15 (0.1)
Borg RPE 0-10 (10=maximal), median (IQR)	10 (9 to 10)
Peak heart rate, beats, min	163 (13)
Percent of predicted peak heart rate (220-age)	100 (7)
Post-exercise blood lactate, mmol/L	9.5 (7 to 12.1) $
Ventilatory reserve, %, mean (SD)	23 (12)

$ *n*=53; values ≥20 mmol/l omitted. RPE: Rating of Perceived Exertion. VCO_2: Volume of carbon dioxide production. VO_2: Volume of oxygen uptake. VO_{2peak}: Peak oxygen uptake.

Figure S1. ROC curves evaluating the ability of eCRF models to predict ≥3.5 mL/kg/min improvement in $VO_{2peak.}$ from baseline to 3 months.

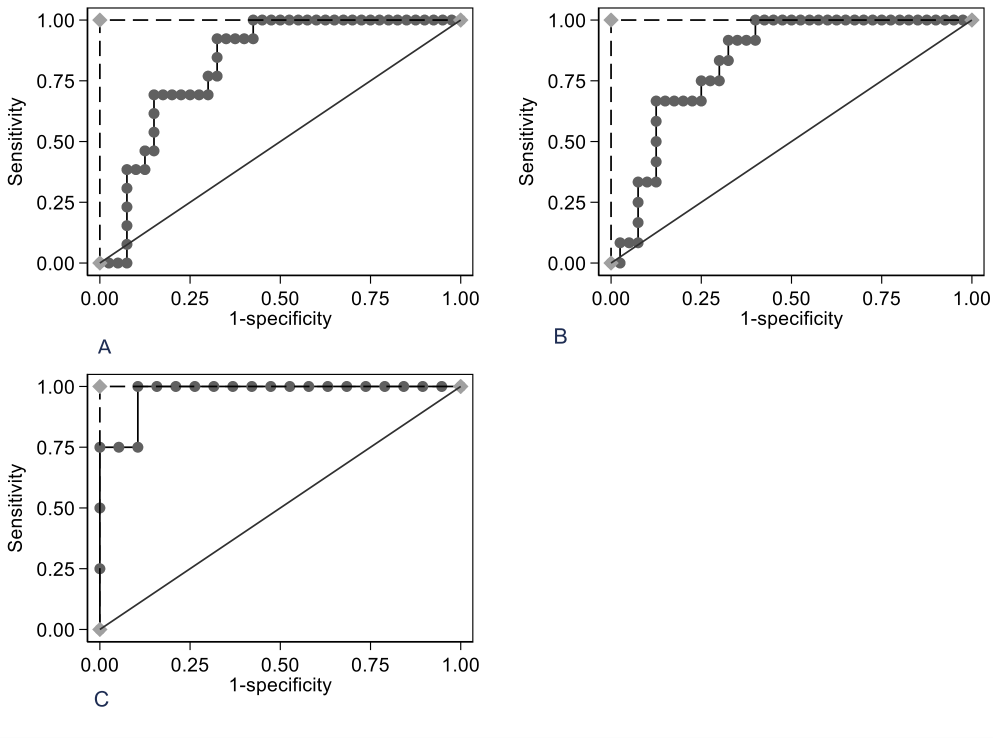

Upper left panel: (**A**) $eCRF_{GEN}$, *n*= 53, AUC 0.82. Upper right panel (**B**): $eCRF_{ALT}$, *n*=52, AUC: 0.83. Bottom left panel (**C**): $eCRF_{PGA}$, *n*=23, AUC: 0.97. AUC: Area under the curve, $eCRF_{ALT}$: Alternative Rheumatoid Arthritis-specific eCRF model, $eCRF_{GEN}$: Estimated cardiorespiratory fitness, generic model, $eCRF_{PGA}$: Rheumatoid arthritis-specific eCRF model. VO_{2peak}: Peak oxygen uptake, ROC: Receiver operating curve.
